# Allogeneic Hematopoietic Stem Cell Transplantation for Acute Myeloid Leukemia With a Germline *DDX41* Mutation

**DOI:** 10.1155/2024/4611649

**Published:** 2024-11-01

**Authors:** Shuro Yoshida, Yuichiro Semba, Shuichiro Takashima, Masanori Kadowaki, Ken Takase, Takahiro Maeda, Koichi Akashi, Hiromi Iwasaki

**Affiliations:** ^1^Department of Hematology, National Hospital Organization Kyushu Medical Center, Fukuoka, Japan; ^2^Department of Medicine and Biosystemic Science, Kyushu University Graduate School of Medical Sciences, Fukuoka, Japan; ^3^Division of Precision Medicine, Kyushu University Graduate School of Medical Sciences, Fukuoka, Japan

**Keywords:** allogeneic hematopoietic stem cell transplantation, AML, *DDX41*, prognosis

## Abstract

According to the 2016 World Health Organization classification, a germline DEAD-box helicase 41 gene (*DDX41*) mutation with myeloid neoplasms has been newly classified. The clinical course of acute myeloid leukemia (AML) with a germline *DDX41* mutation has not yet been clarified. In the early phase, this condition is slowly progressive, the rate of remission induction is high, and the prognosis is good. On the other hand, in the late phase, the gradual relapse rate increases and the ultimate prognosis can be poor. Currently, clear guidance on the indication for allogeneic hematopoietic stem cell transplantation (allogeneic HSCT) for AML with a germline *DDX41* mutation has not been yet provided. However, we consider that allogeneic HSCT should be performed in patients who are eligible for allogeneic HSCT for germline *DDX41* mutations in AML to overcome poor relapse-free survival, referring to previous relevant papers. We report a 49-year-old patient who had pancytopenia and was finally diagnosed with a germline *DDX41* mutation and AML. We decided to perform allogeneic HSCT. On day 68, he was complicated by acute graft versus host disease, gut stage 1, grade II, and was started on prednisolone 0.2 mg/kg. He recovered quickly and has been currently alive without symptoms of graft versus host disease for almost 2 years. Regarding donor search for allogeneic HSCT for AML with a germline *DDX41* mutation, it is essential to ensure that the donor must be negative for this mutation when the donor is a family donor. If the related donor has a positive mutation, which can cause the development of donor-derived leukemia, allogeneic HSCT should performed from an unrelated donor.

## 1. Introduction

According to the World Health Organization in 2016, myeloid neoplasm with germline predisposition was newly classified as a distinct subgroup. In this subgroup, the germline DEAD-box helicase 41 gene (*DDX41*) is one of the germline predispositions [1]. Several reports related to this disease have been published, and its clinical aspects have gradually been understood. However, there is no clear guidance for the indication of allogeneic hematopoietic stem cell transplantation (allogeneic HSCT) for AML with a germline *DDX41* mutation. A recent report suggested that if patients with *DDX41* germline mutation with acute myeloid leukemia (AML) do not receive allogeneic HSCT at first complete remission (1CR), most of them would eventually relapse [2]. We consider that these patients should have allogeneic HSCT performed. Here, we report the case of a patient with AML and a germline *DDX41* mutation who received allogeneic HSCT.

## 2. Case Presentation

**Figure 1:**
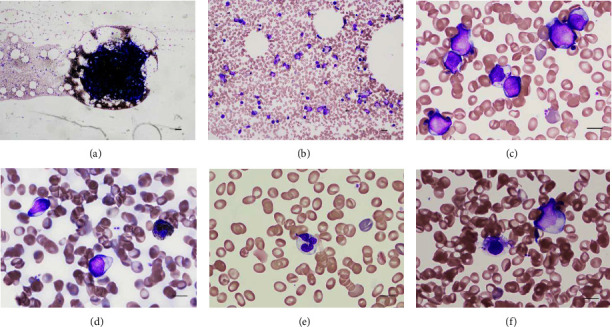
Bone marrow smear findings. (a–c, e, f) The sections were stained with May–Giemsa. (a) × 40. Bar = 100 *μ*m; (b) × 200. Bar = 20 *μ*m; (c, e, f) × 1000. Bar = 10 *μ*m; (d) Myeloperoxidase stain, × 1000.

The patient was a 49-year-old man. In mid-June, he had pancytopenia, a white blood cell count of 2000/*μ*L, a hemoglobin concentration of 12.5 g/dL, and a platelet count of 90,000/*μ*L in a company physical examination. In early December, he visited his local doctor and discovered that his pancytopenia was progressive. In mid-December, he was referred to our hospital department. His white blood cell count was 1400/*μ*L, neutrophils were 25%, blasts were 0.5%, hemoglobin concentration was 9.1 g/dL, and platelet count was 49,000/*μ*L. No disseminated intravascular coagulation was observed. His lactate dehydrogenase concentration was within the normal range at 188 IU/L, and the C-reactive protein concentration was only slightly elevated at 0.25 mg/dL. No other biochemical tests showed abnormalities. A physical examination did not show particular findings, except for obesity, with a height of 166 cm and body weight of 97.8 kg. There was no medical or family history of note. A bone marrow examination showed normocellularity (Figures 1(a) and 1(b)), and the percentage of blasts increased to 34.8%. The blasts were small to medium in size, a round nucleus, delicately reticulated nuclear chromatin, some with distinct nucleoli, a basophilic cytoplasm (Figure 1(c)), and negative myeloperoxidase staining (Figure 1(d)). We also observed granulocytes with reduced granules (Figure 1(e)), erythrocytes with irregular karyotype, and low numbers of micro- and small megakaryocytes (Figure 1(f)). Flow cytometry analysis showed that the percentage of cells in the CD45dim blast gate increased to 10.6% and the surface antigens of these cells were positive for CD13, CD33, CD34, CD117, myeloperoxidase, and HLA-DR, and negative for TdT, cyCD79a, and cyCD3. We diagnosed the patient with AML with minimal differentiation or AML with myelodysplasia-related changes, taking into account the previous history of pancytopenia.

**Figure 2:**
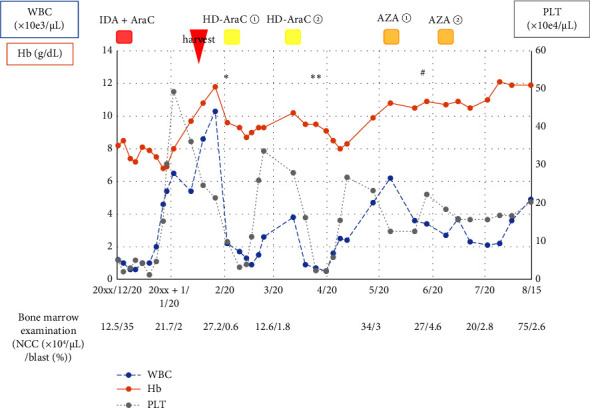
Clinical course from the date of diagnosis to before allogeneic hematopoietic stem cell transplantation. ⁣^∗^DISCAVar analysis in the bone marrow of the patient; ⁣^∗∗^DISCAVar analysis in the buccal mucosa of the patient; ^#^DISCAVar analysis in the buccal mucosa of the older brother of the patient. AZA, azacitidine; HD-AraC, high-dose cytarabine; IDA + AraC, idarubicin + cytarabine; NCC, nucleated cell count.

On 20 December, induction therapy with idarubicin and cytarabine was started (Figure 2). Later, a chromosomal examination of bone marrow cells showed 46XY with normal karyotype. A chimera screening test showed no fusion protein. We judged the patient's AML to pose an intermediate risk. He had one brother. We performed HLA typing and found that they were HLA-identical siblings. We started to prepare the brother as a donor candidate. One month after induction therapy, complete remission was confirmed by bone marrow examination. He started the first consolidation therapy with high-dose cytarabine in mid-February of the following year. A sufficient amount of CD34-positive peripheral blood hematopoietic stem cells was harvested from the brother in early February. We planned to perform allogeneic HSCT after the first consolidation. After 5 days of consolidation therapy, we received a DISCAVar report, a panel test for 398 genes frequently mutated in hematologic malignancies conducted by Kyushu University, using his bone marrow sample at onset. The report revealed the presence of mutation of *DDX41* (NM_016222.3) in p.R525H (c.G1574A, variant allele frequency [VAF] 0.22) and p.P499fs (c.1496dupC, VAF 0.47). We strongly suspected that his AML was a myeloid neoplasm with a germline *DDX41* mutation. Further analysis was performed using his buccal mucosa, and we found a mutation *DDX41* at p.P499fs (VAF 0.48). His AML was confirmed as a myeloid neoplasm with a germline *DDX41* mutation.

**Figure 3:**
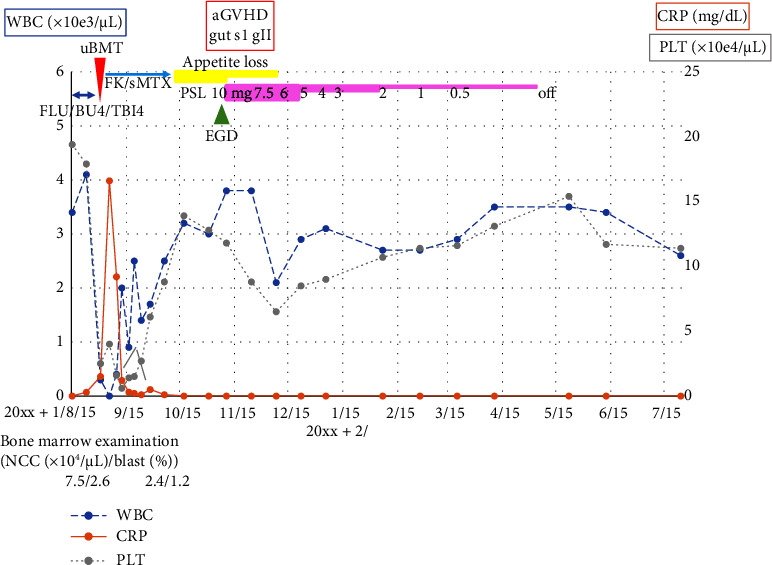
Clinical course after allogeneic hematopoietic stem cell transplantation. aGVHD, acute graft versus host disease; BU, busulfan; EGD, esophagogastroduodenoscopy; FK, tacrolimus; FLU, fludarabine; NCC, nucleated cell count; PSL, prednisolone; sMTX, short-term methotrexate; TBI, total body irradiation; uBMT, unrelated bone marrow transplantation.

We decided to continue performing allogeneic HSCT for the patient. We had to reconsider the donor for his allogeneic HSCT. We had already collected stem cells from his brother, so we needed to check whether the brother had the patient's germline mutation. Previous reports showed that, when using a related donor who had the same germline *DDX41* mutation, donor cell leukemia occurred after reconstitution in the recipient [3–5]. We tested the germline predisposition of the brother using buccal mucosa after informed consent, and he also had a *DDX41* mutation at p.P499fs (VAF 0.5). We explained these results to the patient and his brother. We started to organize an unrelated donor from the Japan Marrow Donor Program. A suitable donor was found in mid-July. After the patient received a conditioning regimen of fludarabine/busulfan/total body irradiation (4 Gy) at the end of August, allogeneic HSCT was performed at 1CR (Figure 3). On day 16, neutrophil cell engraftment was performed, and the platelet count recovered to > 20,000/*μ*L on day 30. His general condition then improved gradually, and he was discharged on day 56. On day 52, his appetite decreased and his symptoms persisted. On day 68, an endoscopic examination of the upper digestive canal was performed. The endoscopic and histopathological findings were not remarkable, but we concluded that his symptoms were caused by acute graft versus host disease (GVHD) from the exclusionary diagnosis. The level of GVHD was gut stage 1, grade II, and prednisolone was started at 0.2 mg/kg. His condition gradually improved, and the dose of prednisolone was gradually decreased. Currently, he is alive with no relapse or GVHD symptoms without prednisolone for almost 2 years. Tacrolimus trough concentrations have also been reduced to below sensitivity 1 year after allogeneic HSCT.

## 3. Discussion

Not so long ago, information on gene abnormalities of hematopoietic disease was not possible. Without the knowledge of a germline mutation *DDX41* in this patient and his brother, the patient would have received a related HLA-identical HSCT at 1CR, and it would likely have relapsed with donor cell leukemia [3–6]. Currently, we can perform a comprehensive genetic analysis and look for genetic abnormalities in patient's leukemogenesis if we have a chance [1, 2, 7]. As in the present case, we believe that from now on, de novo patients with AML who are eligible for allogeneic HSCT should undergo disease-related genetic testing whenever possible.

*DDX41* mutations in hematopoietic tumors were first identified by a research group in the United States in 2015 [8]. The gene is found in a chromosomal region in 5q35.3 and encodes an ATP-dependent RNA helicase. It is believed to be involved in RNA splicing and also plays a role in coordinating transcription elongation of RNA splicing [9, 10]. An interesting feature of this mutation is that a certain percentage of the mutations are germline mutations. In the US study cohort, mainly p.D140fs mutations were identified as germline mutations. On the other hand, somatic mutations are reported to be mainly concentrated in the p.R525H mutation, but the relationship between these mutations is not yet known in detail [2]. Although the *DDX41* gene is characterized by the development of familial myeloid neoplasms, the mean age of onset is 60 years, which is no different from that of unmutated myelodysplastic syndrome (MDS) and AML. This is an interesting characteristic that distinguishes it from other inherited AML [8, 9, 11, 12]. By searching for these germline mutations, we can detect individuals who are approximately 10-fold more likely to develop myeloid neoplasm in the future [12–14]. A recent report showed the cumulative incidence of myeloid neoplasms in a cohort of 384 first-degree relatives (kin cohort) of cases of myeloid neoplasms of the *DDX41* mutant. In this report, the penetration of pathogenic germline mutants of *DDX41* was negligible at an age < 40 years, but increased rapidly thereafter, reaching 49% at 90 years [12]. The incidence of *DDX41* germline mutations in AML is unexpectedly high, with a percentage of approximately 2.4%–6.1% [8, 15, 16].

If a *DDX41* mutation is identified in patients with AML, their treatment needs to be changed. First, the indication of allogeneic HSCT for these patients should be reconsidered. Currently, clear guidance for the indication of allogeneic HSCT for AML with a germline *DDX41* mutation has not yet been shown. Previous reports showed that the myeloid neoplasm with the germline *DDX41* mutation was slowly progressive and the rate of remission after induction therapy for high-risk MDS/AML with germline *DDX41* mutation was high, with a good prognosis. Therefore, these reports suggested that allogeneic HSCT was not needed in 1CR [11, 17]. However, a recent study in patients with AML and a *DDX41* germline mutation who required intensive chemotherapy showed that, after approximately 1 year from onset, relapse increased. Additionally, approximately 90% of patients relapsed 4 years after their diagnosis if they did not receive allogeneic HSCT at 1CR [2]. The median follow-up in the two previous reports [11, 17] was 2.8 and 1.7 years, respectively, and that of the recent study [2] was 4 years. Therefore, there could be a difference in the endpoint between the studies. The backgrounds of the patients were also different between these studies. The two earlier studies [11, 17] reported patients with mixed MDS and AML, while another study [2] reported only AML that required intense chemotherapy. In cases such as the present case, we consider that the latter study [2] should be consulted to determine the course of future treatment. Furthermore, regarding donor search for allogeneic HSCT, it is essential to confirm first that the donor must be negative for this mutation when the donor is a family donor. If the related donor has a positive mutation, which can cause the development of donor-derived leukemia, allogeneic HSCT should performed from an unrelated donor [3–5]. Regarding the conditioning regimen, one report stated that nonrelapse mortality (NRM) increased in AML with a germline *DDX41* mutation compared to AML without that mutation during allogeneic HSCT [2]. Another article also said that patients with AML with the *DDX41* mutation undergoing allogeneic HSCT had a high rate of NRM (1 year 45%) [18]. Currently, there are no well-defined guidelines for appropriate conditioning regimens for allogeneic HSCT for inherited hematologic malignancies, including *DDX41* mutations. However, if mutations are present in DNA damage response genes in germ cell lines or the patient's disease is Fanconi anemia or Li-Fraumeni syndrome with a predisposition to carcinogenesis, a high-dose total body irradiation regimen should be avoided due to the risk of secondary cancers [6, 19]. Some studies have shown that patients with the *DDX41* mutation tend to have solid tumors [11, 20, 21]. Therefore, to avoid NRM and secondary cancers, too much conditioning might be avoided. Regarding GVHD, a previous study reported that the rates of grade III–IV severe acute GVHD and moderate-to-severe chronic GVHD increased in myeloid neoplasms with *DDX41* mutation in allogeneic HSCT [22]. This study suggests that strong conditioning should be employed with caution to avoid GVHD exacerbations. In MDS mutated with the *DDX41* germline, unlike AML with a *DDX41* mutation, continuation with careful observation without treatment shows good results when cytopenia is minimal and may be a good option [23]. The indications for allogeneic HSCT in patients with *DDX41*-mutated MDS may differ from those for *DDX41*-mutated AML. We hope that further analysis will allow for a better treatment of the myeloid neoplasm related to *DDX41* mutation [9, 12].

## 4. Conclusions

In conclusion, we present a 49-year-old patient with AML with a germline *DDX41* mutation. He received allogeneic HSCT by unrelated bone marrow transplantation after canceling the donor candidate, who was the HLA-identical brother, due to his *DDX41* preposition, and is currently well alive. Referring to related articles, we recommend using allogeneic HSCT for a germline *DDX41* mutation in patients with AML if these patients are eligible to overcome poor disease-free survival. Disease-specific precautions should be taken in the case of allogeneic HSCT for AML with a germline *DDX41* mutation.

## Data Availability

The data can be made available from the corresponding author on a reasonable request.
